# Image analysis for bright-field HER2 in situ hybridization: validation for clinical use

**DOI:** 10.1007/s00428-024-03889-3

**Published:** 2024-08-07

**Authors:** Ruoyu Shi, João Correia Pinto, Ivan Rienda, Peter Caie, Catarina Eloy, António Polónia

**Affiliations:** 1 Department of Anatomical Pathology, Singapore General Hospital, Singapore, Singapore; 2 Department of Pathology and Laboratory Medicine, Kandang Kerbau Women´S and Children´S Hospital, Singapore, Singapore; 3 Department of Pathology, Ipatimup, Porto, Portugal; 4 Department of Pathology, Hospital Universitari I Politècnic La Fe, Valencia, Spain; 5 Indica Labs, Albuquerque, NM USA; 6 Faculty of Medicine, University of Porto, Porto, Portugal; 7 Escola de Medicina e Ciências Biomédicas, Universidade Fernando Pessoa, Porto, Portugal

**Keywords:** HER2, In situ hybridization, Quantitative image analysis, Whole slide image

## Abstract

The aim of the present study was to develop and validate a quantitative image analysis (IA) algorithm to aid pathologists in assessing bright-field HER2 in situ hybridization (ISH) tests in solid cancers. A cohort of 80 sequential cases (40 HER2-negative and 40 HER2-positive) were evaluated for HER2 gene amplification with bright-field ISH. We developed an IA algorithm using the ISH Module from HALO software to automatically quantify HER2 and CEP17 copy numbers per cell as well as the HER2/CEP17 ratio. We observed a high correlation of HER2/CEP17 ratio, an average of HER2 and CEP17 copy number per cell between visual and IA quantification (Pearson’s correlation coefficient of 0.842, 0.916, and 0.765, respectively). IA was able to count from 124 cells to 47,044 cells (median of 5565 cells). The margin of error for the visual quantification of the HER2/CEP17 ratio and of the average of HER2 copy number per cell decreased from a median of 0.23 to 0.02 and from a median of 0.49 to 0.04, respectively, in IA. Curve estimation regression models showed that a minimum of 469 or 953 invasive cancer cells per case is needed to reach an average margin of error below 0.1 for the HER2/CEP17 ratio or for the average of HER2 copy number per cell, respectively. Lastly, on average, a case took 212.1 s to execute the IA, which means that it evaluates about 130 cells/s and requires 6.7 s/mm^2^. The concordance of the IA software with the visual scoring was 95%, with a sensitivity of 90% and a specificity of 100%. All four discordant cases were able to achieve concordant results after the region of interest adjustment. In conclusion, this validation study underscores the usefulness of IA in HER2 ISH testing, displaying excellent concordance with visual scoring and significantly reducing margins of error.

**Supplementary Information:**

The online version contains supplementary material available at 10.1007/s00428-024-03889-3.

## Introduction

Breast cancer is the most common cancer and the leading cause of global cancer mortality among women [1]. The overexpression/amplification of human erb-b2 receptor tyrosine kinase 2 (ERBB2 or HER2), a member of the epidermal growth factor receptor family, is observed in 15–20% of breast cancers and is predictive in the treatment response of its targeted therapeutic agents [2–4]. Hence, the determination of HER2 positivity, by either overexpression or gene amplification, is fundamental to current treatment decision-making. In current practice, HER2 status is determined through immunohistochemical and/or in situ hybridization (ISH) assays (fluorescence or bright-field). The 2023 ASCO/CAP (American Society of Clinical Oncology/College of American Pathologists) guideline recommends evaluating a minimum of 20 non-overlapping nuclei of tumor cells in two separate areas of invasive cancer when reporting the HER2 ISH test [5]. Previously, we have demonstrated that increasing the number of evaluated cells from 20 to 60 cells leads to a significant increase in both intra- and interobserver concordance rates [6]. Moreover, we have also demonstrated that the margins of error in HER2 ISH test are very high, even when evaluating 100 cells, which is time-consuming and so rarely performed in clinical practice [7].

Over the past several years, advancements in whole slide image (WSI), computational power, and data storage have brought digital pathology into routine diagnostic practice [8, 9]. Based on this, leveraging WSI and quantitative image analysis (IA) algorithms for the detection and quantification of HER2 ISH slides could overcome some of the limitations of visual scoring, increasing the number of cells scored and decreasing the margins of error, thus providing unbiased and standardized scores.

In this study, we aim to develop and clinically validate an IA software algorithm as a clinical adjunctive aid for pathologists to assess HER2 amplification in solid cancers.

## Materials and methods

### Case selection

A cohort of 80 sequential cases (40 HER2-negative and 40 HER2-positive) with an equivocal HER2 result by IHC (score of 2 +) was retrieved from the digital archive of Ipatimup Diagnostics (Porto, Portugal). The cases were scanned with the Pannoramic® 1000 (3DHistech Ltd.®, Budapest, Hungary) at × 40 magnification (0.121 µm pixel size) and saved in the MIRAX file format (.mrxs). HER2-negative cases were selected between January and February 2023, and the HER2-positive cases were selected between January and July 2023. The cases included formalin-fixed paraffin-embedded (FFPE) needle core biopsies (NCBs) and surgical excision specimens (SESs) referred to our institution (national reference center for HER2 ISH) for an evaluation of HER2 amplification with bright-field ISH. A HER2 test by IHC was performed by the sending institution, and information regarding pre-analytical conditions as well as the antibody used was not available. Cases with HER2 genetic heterogeneity, defined by the 2023 ASCO/CAP guideline as a discrete population of tumor cells with HER2 amplification, were not included [5].

The cohort included 65 needle core biopsies (81.25%) and 15 surgical excision specimens (18.75%), diagnosed in 77 women and in 3 men. The age of the patients ranged from 29 to 83 years old, with a median age at diagnosis of 57 years old. The primary sites of the invasive carcinomas included breast (*n* = 72), stomach (*n* = 3), and endometrium (*n* = 1). The metastatic sites included regional lymph nodes (*n* = 2) and lung (*n* = 2), all from breast cancer primary origin. Cohort characteristics are summarized in Table 1.

**Table 1:** Cohort characteristics

	Number (%)
Sample (NCB/SES)	65 (81.25%)/15 (18.75%)
Gender (female/male)	77 (96.25%)/3 (3.75%)
Age (median [P25–P75])	57 [47–69]
Primary	
Breast carcinoma	72 (90%)
Gastric carcinoma	3 (3.75%)
Endometrium carcinoma	1 (1.25%)
Metastases (from breast)	
Lymph node	2 (2.5%)
Lung	2 (2.5%)

*NCB* needle core biopsy, *SES* surgical excision specimen, *P25* percentile 25, *P75* percentile 75

This study has been performed in accordance with the national regulative law for the handling of biological specimens from tumor banks, being the samples exclusively available for research purposes in retrospective studies, as well as under the international Helsinki Declaration. Ethical approval and informed consent were not required for this study.

### Bright-field in situ hybridization

ISH was performed on 3-μm-thick sections from one FFPE block of each case with dual-hapten, dual-color ISH. The dual-probe assay (VENTANA HER2 Dual ISH DNA Probe Cocktail Assay (catalog number 760–6072); Ventana Medical Systems, Inc., Tucson, AZ, USA), which is Food and Drug Administration–approved, contains a HER2 locus-specific probe (black signal) and a control probe specific for the centromere of chromosome 17 (centromere enumeration probe-CEP17, red signal) that allows detection of HER2 amplification by light microscopy. The entire procedure was carried out on an automated staining system (Ventana BenchMark XT Staining System; Ventana Medical Systems, Inc., Tucson, AZ, USA) according to the manufacturer’s instructions. Appropriated positive and negative controls were used in every slide. Optimal staining consists of an absence of non-specific background staining, distinct nuclear morphology, and clear and specific signals within the nucleus.

### ISH interpretation

The samples were quantified for HER2 amplification by a pathologist (AP or JCP). Corresponding hematoxylin and eosin staining was used for the identification of the invasive component of the tumor, and, whenever available, the IHC slide was used to score in the area with the strongest intensity. Only cells with a minimum of one copy of HER2 and CEP17 each were scored. The number of HER2 signals was estimated in clusters, except for doublets, which were counted as a single signal. The visual evaluation of the samples included scanning the entire ISH slide prior to counting and scoring 20 nuclei, in three different areas, for a total of 60 cells. The breast and gastric cancer cases were classified according to the 2023 and 2016 ASCO/CAP guidelines, respectively [5, 10]. The endometrial cancer case was classified according to the International Society of Gynecological Pathologists [11]. Additionally, all cases were classified with the ISH groups used in breast cancer [5].

### Image analysis (IA)

The IA workstation consists of a 12th Generation Intel® Core™ i9-12900 k 3.20 GHz (Intel®, Santa Clara, CA, USA) processor, 64 GB of RAM (4 GHz), a NVIDIA GeForce RTX™ 3090Ti with 24 GB (NVIDIA®, Santa Clara, CA, USA), and a monitor DELL UltraSharp U2419H (1920 × 1080 resolution, Full HD, 24″).

We developed an IA algorithm using the ISH Module from HALO software (v3.6.4134.193, Indica Labs, Albuquerque, NM, USA) to automatically quantify HER2 and CEP17 copy numbers in bright-field ISH, which we named Computational HER2 for ISH (CHERISH). The nuclear detection and segmentation were performed using an AI algorithm within the HALO software (Nuclei Seg (Plugin)–BF v1.0.0), which was already pretrained and did not require additional training. The imaging features were optimized during the development phase. To exclude non-cancer cells from the analysis, such as stromal or inflammatory cells, we used nuclear size cut-off values, rejecting values below 30 µm^2^. Values above 150 µm^2^ were also excluded because they represented clusters of poorly segmented cells. At the end of the analysis, an output mask is superimposed in the original ISH image showing which cells are being analyzed and how many black and red ISH dots are being detected per cell.

In order to evaluate the performance of the IA algorithm, we executed the following tasks. A subset of 20 cases with adjacent normal tissue was selected, and HER2 and CEP17 copy numbers were quantified in at least 20 nuclei per case. We also confirm the inclusion/non-inclusion of 100 cancer cells and 100 non-cancer cells in 20 cases (10 cells per case). In 20 cases, we measured the time in seconds for running the IA as well as the total area of the analyzed ROI. Lastly, all 80 cancer cases were evaluated for IA by a pathologist (AP or JCP), selecting the tumor area (region of interest, ROI) using freehand annotation. Cases with discordant results were reviewed during common microscopy sessions to determine their causes.

### Statistical analysis

Statistical analyses were performed using the Statistical Package for the Social Sciences (SPSS) version 29.0 for Windows. The Mann–Whitney *U* test (MW), the Wilcoxon test (WC), the Kruskal–Wallis test (KW), and the Pearson correlation coefficient (PCC) were used for the comparison of quantitative variables. The level of significance was set at *P* < 0.05.

The IA software was evaluated using concordance and agreement rates, sensitivity, specificity, negative predictive value (NPV), and positive predictive value (PPV). Agreement rates were evaluated with kappa (*k*) statistics. *k*-Values range between 0 (chance agreement) and 1 (perfect agreement) and were satisfactory if greater than 0.81.

The Altman-Bland analysis was used to assess the relationship between the visual and IA measurements of the HER2/CEP17 ratio, the average of the HER2 copy number, and the average of the CEP17 copy number. The *x*-axis represents the mean of the measurements, and the *y*-axis shows the difference between the measurements for each case. The Altman-Bland plots display the mean difference (solid line) and 95% agreement limits (dashed lines). If there is a high agreement between measurements, the differences are expected to be centered around zero, with a narrow agreement limit.

The margin of error (ME) at a 95% confidence interval (CI) was calculated by multiplying the critical value (1.96) with the standard of error (SE). The standard of error was calculated as the ratio of standard deviation with the squared root of the number of cells analyzed. Curve estimation regression models were used to describe the behavior of the margins of error with the number of invasive cancer cells.

## Results

**Fig. 1:**
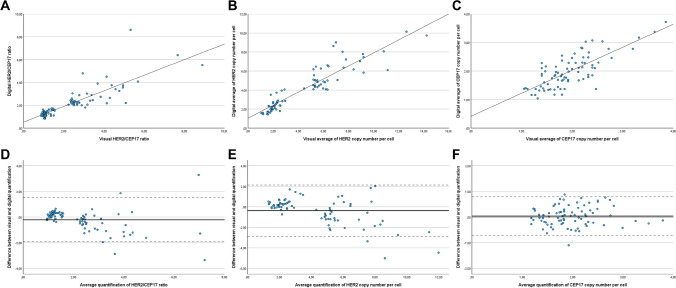
Relationship between visual and digital quantification of HER2/CEP17 ratio (**A**), average of HER2 copy number per cell (**B**), and average of CEP17 copy number per cell (**C**). Altman-Bland analysis of HER2/CEP17 ratio (**D**), average of HER2 copy number per cell (**E**), and average of CEP17 copy number per cell (**F**)

The quantification of the HER2/CEP17 ratio using IA in normal tissue was 0.98, and the average of HER2 and CEP17 copy number per cell was 1.98 and 2.08, respectively. Regarding cancer measurements, we observed a high correlation of HER2/CEP17 ratio, an average of HER2 copy number per cell, and an average of CEP17 copy number per cell between visual and IA quantification (PCC = 0.842, *p* < 0.001; PCC = 0.916, *p* < 0.001; PCC = 0.765, *p* < 0.001; respectively) (Fig. 1A, B, and C, respectively). The quantification of the HER2/CEP17 ratio, of the average of HER2 and CEP17 copy number per cell, was not statistically different between visual and IA measurements (median of 1.89 and 1.77 for the HER2/CEP17 ratio, respectively; *p* = 0.089 WC) (median of 4.82 and 4.07 for the average of HER2 copy number per cell, respectively; *p* = 0.086 WC) (median of 1.85 and 1.89 for the average of CEP17 copy number per cell, respectively; *p* = 0.434 WC) (Table 2). The Altman-Bland analysis showed that the average quantification of the HER2/CEP17 ratio and of the average of HER2 copy number per cell using IA was 0.21 and 0.38 lower than visual quantification, respectively, opposite to the average of CEP17 copy number per cell which was 0.04 higher than visual quantification (*p* = 0.184 KW; Fig. 1D, E, and F, respectively). Finally, the IA algorithm had a cancer cell inclusion sensitivity of 0.64, a specificity of 0.83, and a PPV higher than 0.95 with at least 84% of cancer cellularity (Table S1).

**Table 2:** Quantification of HER2 gene by visual and digital quantification

	Visual	Image analysis	*p*
HER2/CEP17 ratio (median) [P25; P75]	1.89 [1.07; 3.13]	1.77 [1.24; 2.45]	0.089^a^
Average of HER2 copy number per cell (median) [P25; P75]	4.82 [1.98; 6.18]	4.07 [2.25; 5.44]	0.086^a^
Average of CEP17 copy number per cell (median) [P25; P75]	1.85 [1.54; 2.24]	1.89 [1.48; 2.30]	0.434^a^
Number of cells (median) [P25; P75]	60 [60; 60]	5565 [2403.5; 13790.5]	** < 0.001**^**a**^
Margins of error (95% CI) of HER2/CEP17 ratio (median) [P25; P75]	0.23 [0.12; 0.39]	0.02 [0.01; 0.05]	** < 0.001**^**a**^
Margins of error (95% CI) of average of HER2 copy number per cell (median) [P25; P75]	0.49 [0.16; 0.65]	0.04 [0.01; 0.08]	** < 0.001**^**a**^

*P25* percentile 25, *P75* percentile 75, *CI* confidence interval

^a^Wilcoxon test

The bold is significant (*p*<0.001)

IA was able to count from 124 cells to 47,044 cells (median of 5565 cells). The margin of error for the visual quantification of the HER2/CEP17 ratio decreased from a median of 0.23 to 0.02 in IA (*p* < 0.001 WC; Table 2). Additionally, the margin of error for the visual quantification of the HER2/CEP17 ratio was higher in HER2-positive cases compared to HER2-negative cases (median of 0.39 and 0.12, respectively; *p* < 0.001 MW). IA quantification was able to reduce the margin of error of the HER2/CEP17 ratio to a median of 0.05 in HER2-positive cases (*p* < 0.001 WC) and to a median of 0.01 in HER2-negative cases (*p* < 0.001 WC). In the same way, the margin of error for the visual quantification of the average of HER2 copy number per cell decreased from a median of 0.49 to 0.04 in IA (*p* < 0.001 WC; Table 2). Likewise, the margin of error for the visual quantification of the average of HER2 copy number per cell was higher in HER2-positive cases compared to HER2-negative cases (median of 0.65 and 0.16, respectively; *p* < 0.001 MW). IA quantification was also able to reduce the margin of error of the average of HER2 copy number per cell to a median of 0.08 in HER2-positive cases (*p* < 0.001 WC) and to a median of 0.01 in HER2-negative cases (*p* < 0.001 WC). Curve estimation regression models showed that a minimum of 469 or 953 invasive cancer cells per case is needed to reach an average margin of error below 0.1 for HER2/CEP17 ratio (power model (β0 = 3.497; β1 =  − 0.578; *R*^2^ = 0.707); *p* < 0.001) or for the average of HER2 copy number per cell (power model (β0 = 7.479; β1 =  − 0.629; *R*^2^ = 0.634); *p* < 0.001), respectively. In our study, 71 cases (88.75%) had an evaluation of at least 1000 cells using IA. Lastly, on average, a case took 212.1 s (28 to 703 s) to execute the IA, which means that it evaluates about 130 cells/s and requires 6.7 s/mm^2^ (Fig. S1).

**Fig. 2:**
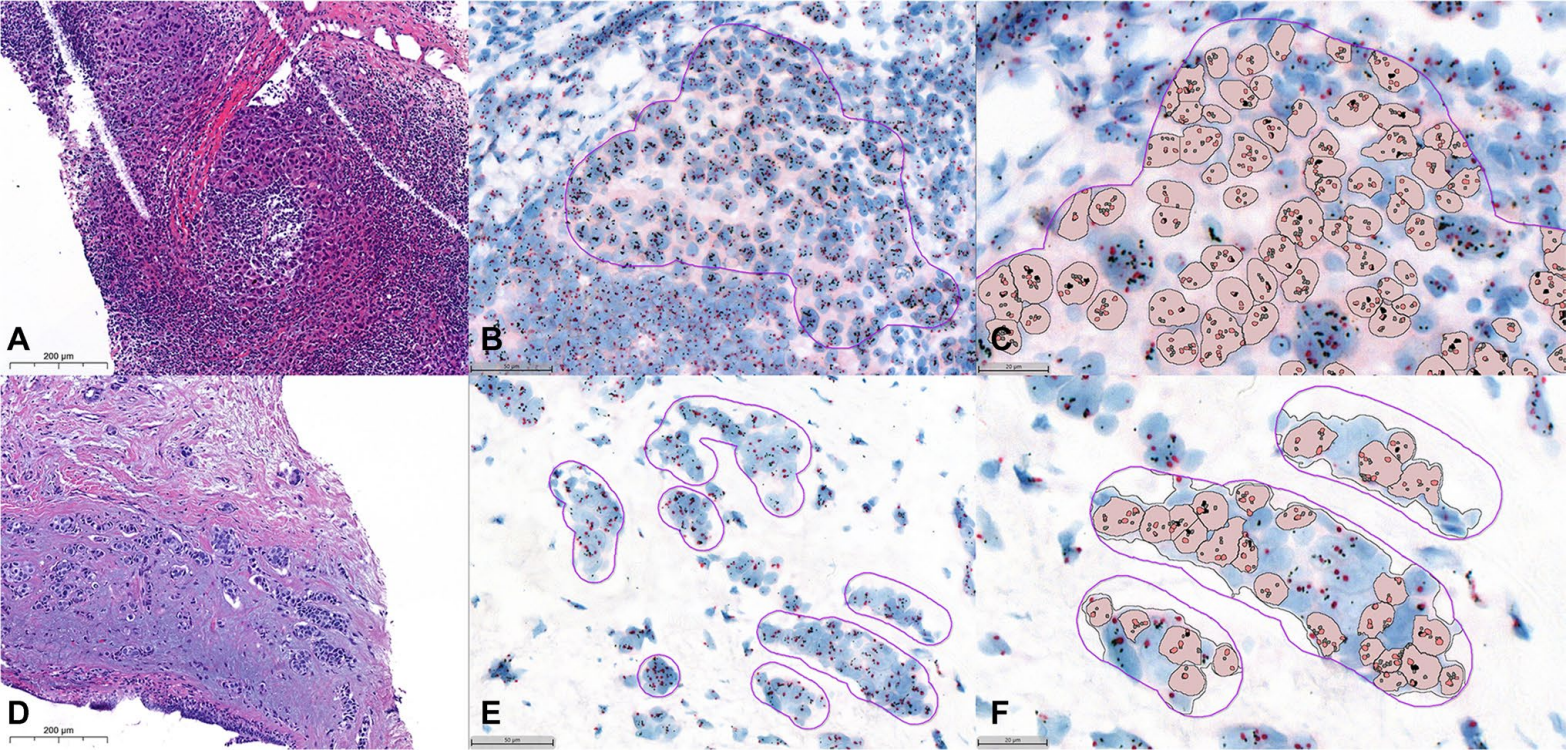
Case #78 (**A**, HE × 40; **B**, ISH × 30; **C**, ISH × 64); case #76 (**D**, HE × 40; **E**, ISH × 30; **F**, ISH × 64)

**Fig. 3:**
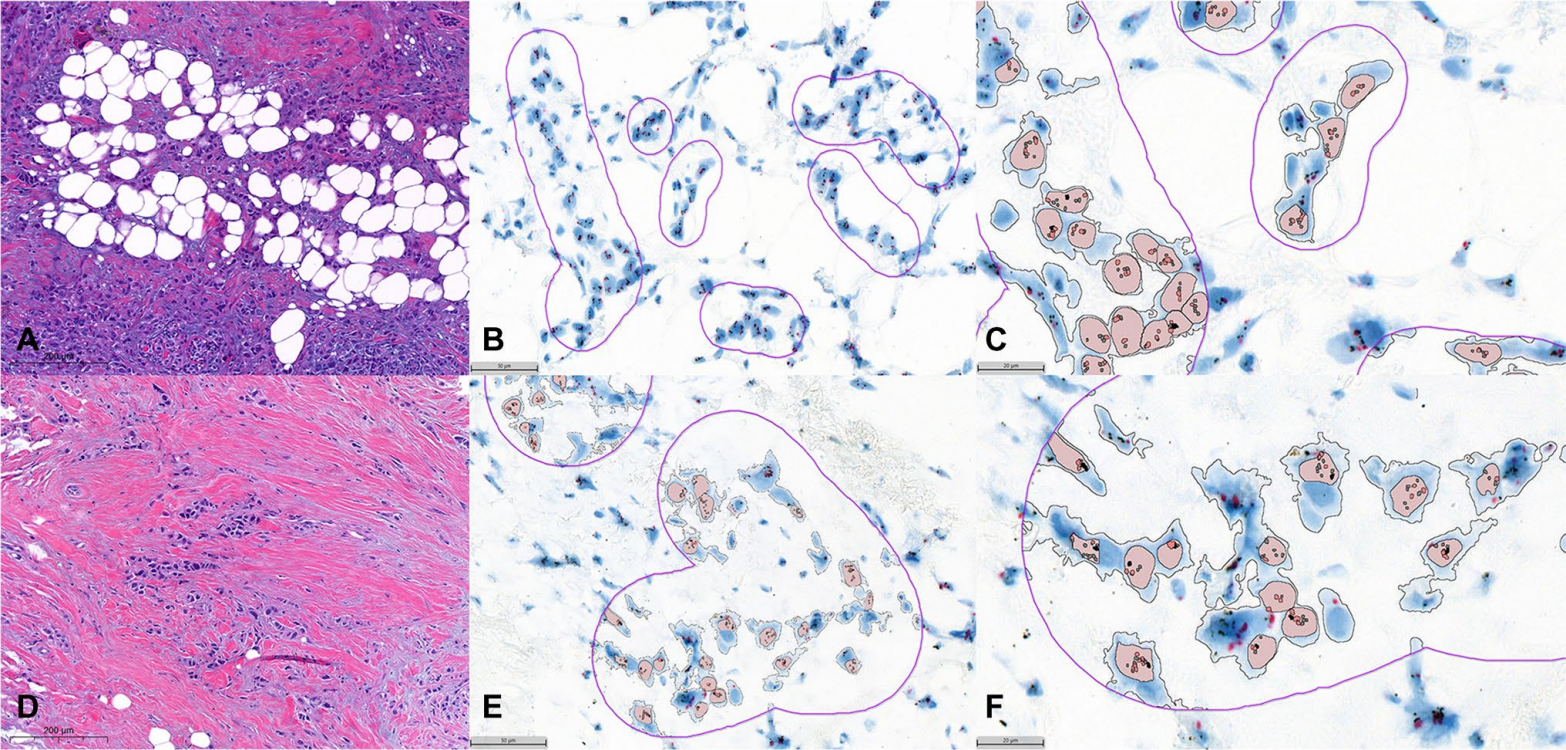
Case #66 (**A**, HE × 40; **B**, ISH × 30; **C**, ISH × 64); case #67 (**D**, × HE 40; **E**, ISH × 30; **F**, ISH × 64)

The comparison of the visual scoring with the IA software showed four discordant cases, which were reviewed during a common microscopy session. The discordant cases consisted of 3 breast carcinomas with low cellularity and an infiltrative pattern of growth (cases #66, #67, and #76) and a lymph node metastasis from breast carcinoma (cases #78), all visually classified as HER2-positive (ISH group 1) and classified by IA as HER2-negative (ISH groups 5, 2, 5 and 4, respectively), with an average of HER2 copy number per cell higher than 3.0 (Table 3, Figs. 2 and 3). The concordance of the IA software with the visual scoring was 95% (*k* = 0.900), with a sensitivity of 90% and a specificity of 100%. Considering a prevalence of 15% of HER2-positive cases, the PPV was 100%, the NPV was 98.27%, and the accuracy of the test was 98.5%. The discordant cases required the selection of smaller ROIs enriched in cancer cells, changing the classification from HER2-negative to HER2-positive (all ISH group 1), concordant with the original visual classification. IA in these cases was able to count from 124 to 218 cells, representing 4 of the 5 cases with fewer tumor cells evaluated. As such, after the pathologist adjustment of ROIs, comparing the visual classification with the IA classification, the algorithm reached an agreement of 100% (*k* = 1.000).

**Table 3:** Discordant cases between visual and digital quantification

	Visual			Initial IA			Final IA		
Case	HER2/CEP17 ratio	HER2 CN	ISH	HER2/CEP17 ratio	HER2 CN	ISH	HER2/CEP17 ratio	HER2 CN	ISH
66	2.43	4.46	1	1.85	3.06	5	2.43	4.16	1
67	2.66	5.32	1	2.42	3.92	2	2.10	4.35	1
76	2.66	5.33	1	1.62	3.72	5	2.04	5.03	1
78	2.35	7.80	1	1.61	4.12	4	2.27	7.22	1

*IA* image analysis, *CN* copy number, *ISH* in situ hybridization group

Additionally, we observed a concordance of ISH group classification between visual and IA of 97.5% (*k* = 0.952), with 2 discordant cases (Table S2). The discordances were observed between groups 1 and 3 (case #40) and between groups 5 and 4 (case #17). Case #40 was a breast cancer originally classified as ISH group 1 with HER2/CEP17 ratio of 3.06 and an average of HER2 copy number of 11.12, classified by IA as ISH group 3 with HER2/CEP17 ratio of 1.80 and an average of HER2 copy number of 6.10. Case #17 was a lung metastasis from breast cancer originally classified as ISH group 5 with HER2/CEP17 ratio of 1.25 and average of HER2 copy number of 2.90, classified by IA as ISH group 4 with HER2/CEP17 ratio of 1.63 and average of HER2 copy number of 4.06. The non-breast cancer cases (gastric and endometrium) had a perfect concordance of ISH groups between visual and IA classification (Table S3).

## Discussion

The evaluation of HER2 bright-field ISH is traditionally performed under an optical microscope by visually counting black and red dots in at least 20 cells in two separate areas of invasive cancer, which is time-consuming, laborious, and tedious and suffers from interobserver variability [6, 12–14]. We developed and validated an IA algorithm to automatically quantify HER2 and CEP17 copy numbers per cell in bright-field ISH whole slide images, establishing the HER2 amplification status in samples with invasive cancer. The IA was able to quantify HER2 status across three separate diseased instances and in both primary and metastatic tissue. This laboratory-developed test (LDT) is intended to be used as a tool to assist pathologists in standardizing the routine time-consuming task of HER2 gene quantification.

In breast cancer, HER2 gene quantification through IA has already been used in fluorescence ISH (FISH), showing good concordance between visual quantification (reference method) [15–17]. The quantification of HER2 and CEP17 copy numbers per cell in normal tissue using CHERISH software was similar to the biologically expected values of 2.0 copies per nucleus and a HER2/CEP17 ratio of 1.0, suggesting an appropriate measurement methodology with optimized image analysis parameters. Notably, in our study, we observed a small trend for decreased quantification of the HER2/CEP17 ratio and of the average of HER2 copy numbers per cancer cell using IA quantification and an opposite trend for the average of CEP17 copy number. Previously, a tendency for a decreased average of both HER2 and CEP17 copy numbers per cell using IA quantification on FISH has been reported [18]. The inconvenience of the IA on the FISH technique is that it requires software exposure settings to be adjusted regularly to correct for aging light sources or after the change of fluorescence excitation bulbs, after which image quality improves significantly. Using CHERISH, the average difference between visual and IA quantification was lower than previously reported (− 0.38 versus − 0.56 signals and + 0.04 versus − 0.13 signals for the average of HER2 and CEP17 copy number per cell, respectively) [18]. The data reflects a limitation of the algorithm in selecting cancer cells, rejecting non-cancer cells based on size cutoffs. The inclusion of non-cancer cells decreases HER2 quantification, affecting both the HER2/CEP17 ratio and the average of HER2 copy number per cell, and a high PPV is only achieved with high cancer cellularity (about 85%). This highlights the importance of the pathologist in selecting a proper ROI enriched in cancer cells for accurate HER2 quantification using IA. Importantly, we observed a high correlation of quantification of the HER2/CEP17 ratio and the average of HER2 and CEP17 copy number per cell between visual and IA measurements of cancer cases without statistically significant differences, confirming equivalency between methodologies.

ISH assays are quantitative methods used to measure the number of HER2 and CEP17 copies per nucleus, enabling precise quantification of the HER2 gene in cancer tissue. Despite the potential of IA to quantify thousands of cells, existing approaches have been focused on automatically counting the same number of cells as visual assessment, not fully exploiting the technology’s capabilities [15–17, 19, 20]. Previous studies, including our own, have demonstrated the importance of analyzing a high number of cells, leading to increased intraobserver and interobserver concordance rates, as well as a notable reduction in the margins of error for HER2/CEP17 ratio and average of HER2 and CEP17 copy number measurements [6, 7]. In our study, approximately 90% of cases contained a sufficient number of cancer cells to reach an average margin of error below 0.1 for both the HER2/CEP17 ratio and the average of HER2 copy number per cell, all achieved within a clinically acceptable analysis time of under 4 min. Hopefully, with the general adoption of these quantitative methods, the precise quantification of the HER2 gene, and the inclusion of its margins of error in the report, could eventually allow for more personalized management of these patients.

The uPath HER2 Dual ISH IA algorithm (Roche Ventana, Tucson, AZ, USA) was designed to assist pathologists in HER2 gene quantification using bright-field ISH in breast cancer [20]. This software highlights areas of HER2 amplification in the form of a heat map, where cells are selected based on the segmentation quality, signal detection per cell, and the likelihood of the cell being a tumor cell. Recent research has indicated that the use of such a tool significantly enhances the interobserver concordance rate of HER2 amplification status, as well as ISH group classification, including less common and more challenging non-classical groups [21]. Non-classical ISH group cases are challenging, representing up to 10% of the cases, and requiring a higher number of cells for evaluation and, preferably, a second independent observation [22]. In our study, we only had one non-classical ISH group case (group 4) that was correctly classified by IA. Nonetheless, two classical ISH group cases (groups 1 and 5) were classified by IA as non-classical ISH group cases (groups 3 and 4, respectively), which did not change the final classification of HER2 status. The reason for this discordance in ISH group classification is probably related to the selection of different areas between visual and IA evaluation. Interestingly, we had already reported that a low proportion of cases (between 5 and 10%) can have areas with different ISH group classifications but the same final HER2 status [7]. These cases represent a form of non-conventional heterogeneity that is usually not measured or reported and may show different clinical behavior compared to homogenous cases, a condition that could be explored in future studies.

The College of American Pathologists (CAP) guideline for quantitative image analysis (QIA) of HER2 requires a minimum of 40 HER2-positive and 40 HER2-negative cases for LDT validation [23]. Although initially established for breast cancer immunohistochemistry, this guideline can be adapted for fluorescence or bright-field ISH. Our study demonstrated an initial concordance of 95% between the IA software and visual scoring, with 100% specificity. However, cases with low cellularity may yield false-negative results, requiring pathologist intervention to ensure evaluation exclusively of cancer cells, thereby avoiding the inclusion of non-cancer cells that distort HER2 and CEP17 copy number per cell averages. Following pathologist correction to select ROIs enriched in cancer cells, IA classification achieved a perfect agreement with visual scoring, highlighting the importance of pathologist expertise and supervision in this analysis. In our experience, this manual correction can be performed in a few minutes, depending on the number of new ROIs which is at the discretion of the pathologist. The software’s output mask can be used to check the correct detection and classification of nuclei, black, and red ISH dots, ensuring precise quantification. While our current study relies on nuclei size cutoffs to include cancer cells, efforts are already being made to develop a cancer classifier based on a neural network using the HALO software to improve the automatic detection of cancer cells.

The implementation of these technologies requires changing the traditional workflow into a digital one, with the generation of routine WSI [24]. Despite the inclusion of cases originating from several pathology laboratories, some limitations of our work are the low representation of non-classical ISH groups, the exclusion of cases with HER2 genetic heterogeneity, and the lack of clinical correlation with the response to target therapy.

In conclusion, this validation study underscores the usefulness of IA in HER2 ISH testing, displaying excellent concordance with visual scoring and significantly reducing margins of error, thus meeting CAP recommendations for clinical validation and integration of QIA in LDTs [23].

## Supplementary Information

Below is the link to the electronic supplementary material.

Supplementary file1 (DOCX 45 KB)

## Data Availability

The datasets generated during and/or analyzed during the current study are available from the corresponding author on reasonable request.
