# Supplementary material for: Image analysis for bright-field HER2 in situ hybridization: validation for clinical use
Source: Virchows Arch. 2024 Aug 7;486(3):541–9. doi: 10.1007/s00428-024-03889-3 (PMC11950096; doi:10.1007/s00428-024-03889-3)

| **Table S1** Positive and negative predictive values according to cancer cellularity | | |
| --- | --- | --- |
| Cancer cellularity | Positive predictive value | Negative predictive value |
| 5 | 16.54 | 97.77 |
| 10 | 29.49 | 95.40 |
| 20 | 48.48 | 90.22 |
| 25 | 55.65 | 87.37 |
| 30 | 61.74 | 84.33 |
| 40 | 71.51 | 77.57 |
| 50 | 79.01 | 69.75 |
| 60 | 84.96 | 60.58 |
| 70 | 89.78 | 49.70 |
| 75 | 91.87 | 43.46 |
| 80 | 93.77 | 36.56 |
| 90 | 97.13 | 20.39 |
| 95 | 98.62 | 10.82 |
|  | | |

| **Table S2** Distribution of cases per ISH group | | | | | | | |
| --- | --- | --- | --- | --- | --- | --- | --- |
|  |  | Image analysis | | | | |  |
|  | Group | 1 | 2 | 3 | 4 | 5 | Total |
| Visual | 1 | 39 | 0 | 1 | 0 | 0 | 40 |
|  | 2 | 0 | 0 | 0 | 0 | 0 | 0 |
|  | 3 | 0 | 0 | 0 | 0 | 0 | 0 |
|  | 4 | 0 | 0 | 0 | 1 | 0 | 1 |
|  | 5 | 0 | 0 | 0 | 1 | 38 | 39 |
|  | Total | 39 | 0 | 1 | 2 | 38 | 80 |

| **Table S3** Quantification of non-breast cancer cases | | | | | | | | | | |
| --- | --- | --- | --- | --- | --- | --- | --- | --- | --- | --- |
| Case | Gender | Age | Sample | IC | Visual | | | Image Analyis | | |
|  |  |  |  |  | HER2 status | HER2/CEP17 ratio | HER2 CN | HER2 status | HER2/CEP17 ratio | HER2 CN |
| 4 | M | 65 | IB | GC | Negative (Group 5) | 1.01 | 2.30 | Negative (Group 5) | 0.88 | 2.69 |
| 9 | M | 47 | IB | GC | Negative (Group 5) | 1.00 | 1.80 | Negative (Group 5) | 1.47 | 3.50 |
| 28 | M | 55 | IB | GC | Negative (Group 5) | 1.02 | 2.18 | Negative (Group 5) | 1.16 | 2.47 |
| 34 | F | 67 | IB | EC | Negative (Group 5) | 0.94 | 1.20 | Negative (Group 5) | 1.06 | 1.46 |
| F, female; M, male; IB, incisional biopsy; CN: copy number; IC, invasive carcinoma; GC, gastric cancer; EC, endometrial cancer. | | | | | | | | | | |

Figure S2


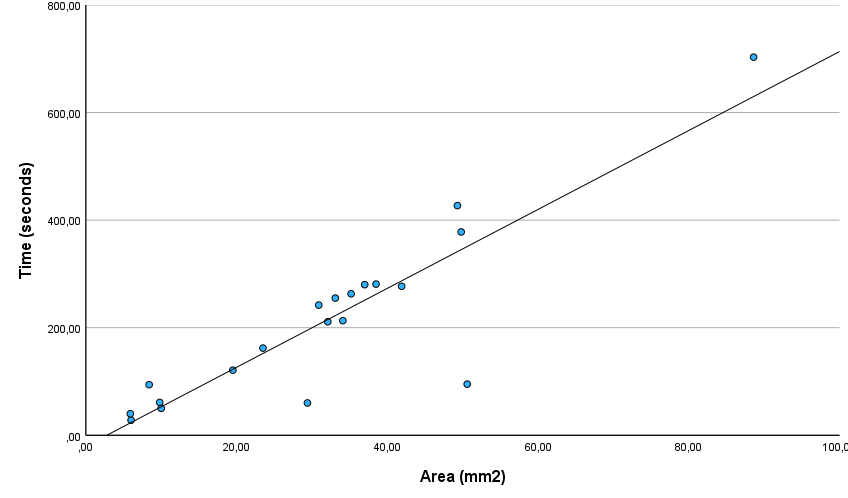

Supplement: Supplementary file 1 — Supplementary file1 (DOCX 45 KB) [file 428_2024_3889_MOESM1_ESM.docx]
